# From provocation to aggression: the neural network

**DOI:** 10.1186/s12868-017-0390-z

**Published:** 2017-10-17

**Authors:** Jonathan Repple, Christina M. Pawliczek, Bianca Voss, Steven Siegel, Frank Schneider, Nils Kohn, Ute Habel

**Affiliations:** 1 Department of Psychiatry, Psychotherapy and Psychosomatics, Medical School, RWTH Aachen University, Pauwelsstraße 30, 52074 Aachen, Germany; 2 JARA BRAIN-Translational Brain Medicine, Pauwelsstraße 30, 52074 Aachen, Germany; 3 Department for Cognitive Neuroscience, Donders Institute for Brain, Cognition and Behaviour, Radboudumc, Kapittelweg 29, 6525 EN Nijmegen, The Netherlands; 4 Department of Psychiatry, University of Pennsylvania, 125 S. 31st Street, Translational Research Building, Philadelphia, PA 19104-4283 USA; 5 Department of Psychiatry, University of Münster, Münster, Germany

**Keywords:** Impulsivity, TAP, PSAP, Neuroimaging, Violence

## Abstract

**Background:**

In-vivo observations of neural processes during human aggressive behavior are difficult to obtain, limiting the number of studies in this area. To address this gap, the present study implemented a social reactive aggression paradigm in 29 healthy men, employing non-violent provocation in a two-player game to elicit aggressive behavior in fMRI settings.

**Results:**

Participants responded more aggressively after high provocation reflected in taking more money from their opponents. Comparing aggression trials after high provocation to those after low provocation revealed activations in neural circuits involved in aggression: the medial prefrontal cortex (mPFC), the orbitofrontal cortex (OFC), the dorsolateral prefrontal cortex (dlPFC), the anterior cingulate cortex (ACC), and the insula. In general, our findings indicate that aggressive behavior activates a complex, widespread brain network, reflecting a cortico-limbic interaction and overlapping with circuits underlying negative emotions and conflicting decision-making. Brain activation during provocation in the OFC was associated with the degree of aggressive behavior in this task.

**Conclusion:**

Therefore, data suggest there is greater susceptibility for provocation, rather than less inhibition of aggressive tendencies, in individuals with higher aggressive responses. This further supports the hypothesis that reactive aggression can be seen as a consequence of provocation of aggressive emotional responses and parallel evaluative regulatory processes mediated mainly by the insula and prefrontal areas (OFC, mPFC, dlPFC, and ACC) respectively.

**Electronic supplementary material:**

The online version of this article (doi:10.1186/s12868-017-0390-z) contains supplementary material, which is available to authorized users.

## Background

Aggression, defined as “any behavior directed toward another individual that is carried out with the intent to cause harm” [1] can further be categorized as reactive versus instrumental aggression. Reactive aggression is impulsive and emotional, whereas instrumental aggression is premeditated, proactive and goal-oriented [1]. Additionally, personal factors such as attitudes, personality traits and genetic disposition as well as situational factors including provocation, frustration, pain and drugs contribute to aggression [1].

Due to the difficulty of inducing overt aggression in an fMRI setting, neuroimaging research on underlying mechanisms of aggressive behavior is a major challenge and there is a paucity of studies assessing acute aggressive behavior in experimental settings. Therefore, theories regarding neural networks for aggression are based primarily on patients with abnormal aggression, animal studies and imaging studies of related psychological constructs (provocation, anger, emotional regulation processes, conflict decision making, and theory-of-mind) [2–4].

Combining results from these different approaches, reactive aggression is seen as a result of an imbalance between the top-down control provided by the orbital frontal cortex (OFC), dorsolateral prefrontal cortex (dlPFC) and anterior cingulate cortex (ACC) and excessive “bottom-up” drives triggered by limbic regions, such as the amygdala and insula [2, 3, 5]. Several theories propose that the network between the limbic system, the OFC, and the dlPFC is responsible for the processing of emotional and goal driven behavior and therefore damage or dysfunction of this network results in emotion regulation difficulties that may lead to impulsive and aggressive behavior [4, 6, 7].

Research has shown that it is possible to induce aggressive behavior in laboratory settings [8–10] and in fMRI [11–13]. Employing a social reactive aggression paradigm in fMRI, increased mPFC activation was observed during retaliation using aversive pneumatic pressure stimuli on the finger [14]. Provocation elicited by use of the Point Aggression Subtraction Task (PSAP) increased relative glucose metabolic rate in anterior, medial, and dorsolateral prefrontal regions, brain regions involved in top-down cognitive control of aggression [15]. Aversive noise as punishment after high provocation [16] led to activations in the dorsal parts of the ACC and bilaterally in the anterior insula during retaliation. Failed motor response inhibition and reactive aggression (measured by the Taylor aggression paradigm, TAP) both activated the anterior insula, suggesting an overarching role of the anterior insula in different aspects of aggression [13, 17]. Chester and DeWall [12] showed that nucleus accumbens activation during provocation predicted retaliatory aggression in a TAP, which points to the involvement of hedonic reward in aggression.

In contrast to those previous studies, the present study aimed to examine (1) the neural processes involved in *non*-*physical* provocation and reactive aggression, and (2) individual differences in the relationship of provocation with aggressive behavior employing associations between behavioral measures, questionnaire scores and neural processes. It employed an fMRI-suitable adaption of the TAP with the added advantage of non-physical provocation comparable to the PSAP, as it reduces the likelihood of movement artifacts, but still employs simple, non-costly aggression (retaliatory punishment) selections. This is in contrast to all recent neuroimaging studies of aggression that employed some form of violent punishment [12, 13, 18]. Based on previous results [14, 16], we hypothesized more aggressive behavior in response to higher provocation. Concerning the neural processes during provocation and subsequent reactive aggression, we based our hypotheses on the previous concept of aggressive behavior as an interaction of limbic regions and cortical control areas. Hence we expected higher activity in the proposed network of amygdala and insula as well as in the mPFC, dlPFC, OFC and the ACC already during provocation, specifically we hypothesized a more active network in high compared to low provocation rounds [2, 3]. Consequently this should also lead to higher activity in such regions during aggression rounds after high versus low provocation.

## Methods

### Subjects

We evaluated 33 right-handed healthy male subjects [mean age = 23.6 years, SD = 3.2; IQ (crystallized intelligence estimation, MWT-B (Multiple Choice Vocabulary Test) [19]): 96.21, SD = 9.19]. Four subjects were excluded due to movement artifact in imaging data. We screened for psychiatric and other illnesses using the Structural Clinical Interview for DSM Disorders (SCID) questionnaire [20, 21].

### Questionnaires

In order to assess trait aggression and emotions related to the paradigm, participants completed the Buss–Perry Aggression Questionnaire (AQ) [22] before testing, as well as the Positive and Negative Affect Schedule (PANAS) [23], the emotional self rating (ESR) [24] and the State-Trait Anger Expression Inventory (STAXI) [25] before and after testing.

### Functional task

During fMRI, a modified version of the Taylor Aggression task, which seeks to elicit aggressive behavior by provocation, was employed. Upon arrival, subjects were told that they were playing a reaction-time game against an opponent, who was supposedly sitting in the next room. In fact, there was no opponent as subjects were playing against a computer. Their task was to press a button faster than their opponent as soon as they saw a soccer ball appearing on the screen. For every winning round subjects were promised an extra 50 cents in addition to their allowance for participation. Before each round, subjects had to decide the amount of money to be subtracted from their opponent if the opponent lost the round. The participants could choose values (multiples of 10) between 0 and 100 cents. It was made explicitly clear that this amount was only subtracted from the opponents’ accounts and not credited to the participants in order to illustrate that this function would only serve as a way to punish the opponents. In total, each participant played 150 rounds. It was predetermined that they would win 60 and lose 90 rounds. The order of rounds was randomized. Of the 90 lost rounds the fake opponent subtracted values between 0 and 100 cents distributed equally over all values and randomized in order to avoid any time effect or supposed strategy. The sequence of the game was the same for all 150 rounds and could be divided into two phases:

1. The punishment selection (aggression) phase: The participants had to choose the amount that was subtracted from the opponent in case of winning.
2. The feedback (provocation) phase: After playing the reaction game, the participants received the results from the trial and learned that they either won (and got 50 cents) or that they lost (in which case the amount that the mock opponent took from them was displayed). Since there is no rational incentive to subtract money from the opponent, but the opponent did this anyway, we operationalized this phase as the provocation phase.

**Fig. 1:**
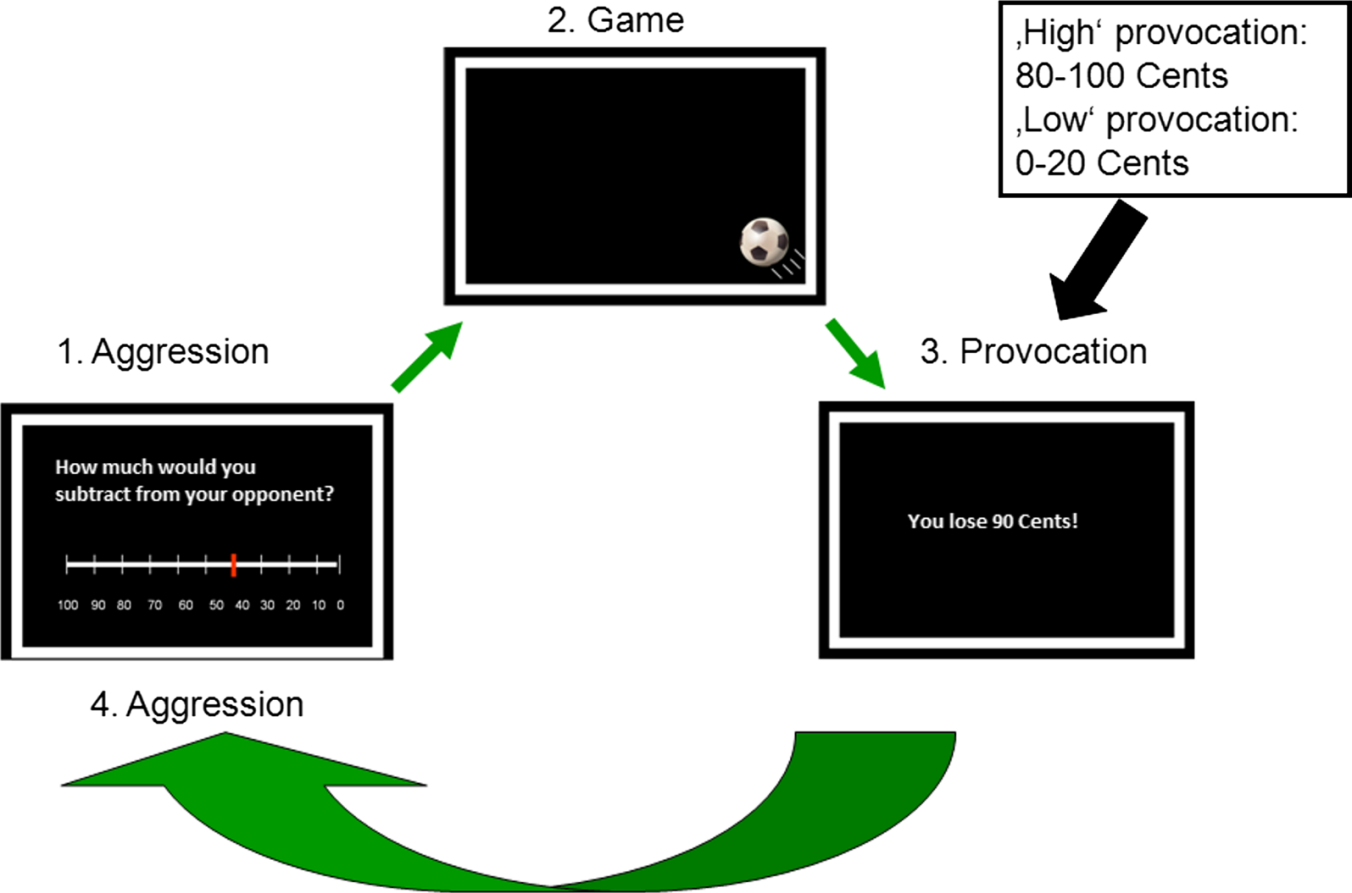
Reactive aggression task: Before the game, subjects chose the punishment for the opponent (1). After the game (2), the participants received the results from the last game (3). After high provocation in this phase, we hypothesized higher aggression in the next aggression phase (4)

The hypothesis is that after rounds of high provocation the participant would in turn choose a higher amount to subtract from the opponent for the next trial, which would resemble our notion of provoked or reactive aggression (Fig. 1).

We defined losing rounds, in which the mock opponent took between 0 and 20 cents from our participant, as “low provocation rounds” and rounds, in which the opponent took 80–100 cents, as “high provocation rounds”. High provocation and low provocation rounds occured on average 25 times each for each participant. Losing rounds, in which he took 30–70 cents, were defined as “intermediate provocation”. “Average aggression” refers to the mean punishment selection score of all the 150 rounds.

The duration of the phases was constant throughout the game with a built-in jitter in the anticipation phase and the inter trial interval (ITI; aggression phase: 4.0 s, jittered anticipation phase: 4.6–4.9 s, game phase: 4.0 s, provocation phase: 3.0 s, jittered ITI: 1.0–2.9 s). The task was programmed using the Presentation software package (Neurobehavioral Systems Inc., Albany, CA, USA). Participants were required to answer by pressing a button using their right index and middle fingers on an MRI-suitable keyboard.

After completing the task, subjects were debriefed with a standardized questionnaire, which was used as a manipulation check. While some subjects questioned the illogical strategy of the opponent, none of them questioned the existence of a human opponent. Therefore we included all subjects that finished the task.

### Behavioral data analysis

The aggression scores were processed with SPSS 21 (SPSS Inc., Chicago, USA). Mean values for average aggression, aggression after high provocation and after low provocation were calculated and analyzed with a paired-sample *t* test. Level of significance was p < 0.05 (two-tailed).

### FMRI data acquisition

Functional imaging was performed on a 3T Trio MR scanner (Siemens Medical Systems, Erlangen, Germany) using echo-planar imaging sensitive to BOLD contrast (T2*, voxel size: 3.0 mm × 3.0 mm × 3.0 mm, 64 × 64 matrix, FoV: 192 mm^2^, 34 slices, gap 0.30 mm, flip angle 77°, TR 2000 ms, TE 28 ms, interleaved order of slice acquisition, slices parallel to anterior–posterior commissure). Before functional testing anatomical images (standardized T1-weighted sequences) were collected (TR = 1900 ms, TE = 2.52 ms, TI = 900 ms, matrix = 256 × 256, 176 slices, FoV: 250 × 250 mm^2^, flip angle = 9°, voxel size = 1 × 1 × 1 mm^3^).

### Analysis and preprocessing

Analyses of functional images were performed with SPM8 (Wellcome Department of Cognitive Neurology, London, UK). Slice time correction, realignment to the mean image, stereotaxic normalization (2.0 mm × 2.0 mm × 2.0 mm), smoothing (8 mm FWHM Gaussian kernel) and high-pass filtering (7.81 mHz) were applied. For segmentation we used an approach called “Unified Segmentation”, a method combining a smooth intensity variation and nonlinear registration with tissue probability maps [26]. All images from the 3 test rounds were discarded (25–26 scans), which also allowed steady-state magnetization. For each participant individual first-level (fixed-effects) analysis was performed, using separate general linear models (GLMs) for each participant. For single subject analyses the different game phases (aggression after high provocation, aggression after low provocation, aggression after intermediate provocation, aggression after win rounds, anticipation phase, game phase, high provocation, intermediate provocation, low provocation, feedback after winning rounds) were included as single regressors. These were constructed by convolution of the event-related delta-functions of each event-type with the canonical hemodynamic response function. The six realignment parameters were included as regressors into the first-level analysis. Data were high-pass filtered with a cutoff-period of 128 s to remove low-frequency drifts from the data. Serial correlations were accounted for by first-order autoregressive model. In order to correct for multiple comparisons we assumed a per voxel probability threshold of p = 0.001 and additionally a cluster-level threshold of p(FWE(family-wise error)) = < 0.05. Anatomical localizations were identified using the Anatomy Toolbox [27] and the WFU Pick Atlas [28] as tools implemented in SPM. For the GLM analysis two contrasts of interest were calculated since they reflected provocation and aggression: High provocation versus low provocation and aggression after high provocation versus aggression after low provocation. Following preceding aggression inducing studies [29], we analyzed the data based on the effects of different levels of provocative feedback in subsequent aggressive decisions (to analyze within-subject differences).

To determine between-subject differences (the influence of individual aggressive response) during the task, we performed a regression analysis using the average aggression score as a covariate (comparable to previous studies [12]). To check for associations with brain activity in the provocation and aggression contrasts (using the high vs. low provocation and aggression after high vs. aggression after low provocation contrast images) we calculated two separate regression analyses.

### Correlational analyses

Correlation analyses were performed between behavioral measures, such as STAXI scores, total score on the AQ and regional activation in the two main contrasts. Specifically, we extracted mean values from all clusters of the main contrasts high versus low provocation and aggression after high provocation versus aggression after low provocation.

## Results

### Behavioral data

Participants selected higher punishments (higher values) after high provocation than after low provocation (high provocation: mean 55.39, SD: 35.37; low provocation: mean 44.37, SD: 33.59; t_28_ = 4.15, p < 0.001). Participants selected significant higher punishment after lose trials than after win trials (punishment after lose trials: mean 48.54, SD: 32.86; punishment after win trials: mean 46.13, SD: 31.40; t_28_ = 7.91, p < 0.001).

The task also successfully induced anger in participants, reflected in higher “Anger” ESR- scores after the game than before [Wilcoxon Signed-rank test: ESR (Anger after): 1.41; ESR (Anger before): 1.10; p = 0.015]. Subjects also showed higher scores on the State-Trait-Anger-Expression-Inventory (Wilcoxon signed-rank test: STAXI post task: 12.00; STAXI pre task: 10.48; p = 0.05). No significant differences on the PANAS scores were found (p > 0.05). There were no significant correlations between behavioral measures (e.g. average aggression) and the Buss–Perry Aggression Questionnaire.

### Functional activation data

#### Whole group

##### Contrast high provocation versus low provocation

Contrasting high versus low provocation yielded activation in the right and left rostral anterior cingulate cortex (rACC), the right and left mPFC, and the right and left thalamus (Table 1).

**Table 1:** Activations for whole sample of all subjects

Brain areas	L/R	X	Y	Z	T	p-value	Cluster size
		(MNI coordinates)					
Aggression after high provocation > aggression after low provocation							
Medial prefrontal cortex (mPFC) + rostral anterior cingulate cortex (rACC) + orbitofrontal cortex (OFC)	R	10	32	54	6.48	<.001	5323
Anterior angular gyrus (part of inferior parietal gyrus)	R	52	– 54	40	4.64	< .001	668
Insula	L	– 30	20	– 18	5.44	< .001	397
Dorsolateral prefrontal cortex (dlPFC) + ventrolateral prefrontal cortex (vlPFC)	R	56	28	20	5.57	< .001	277
Aggression after low provocation > aggression after high provocation							
No suprathreshold activations							
High provocation > low provocation							
rACC + mPFC	R/L	14	40	2	5.54	< .001	819
Thalamus	R/L	10	2	– 2	5.60	< .001	792
Low provocation > high provocation							
No suprathreshold activations							

Activation cluster of all subjects, p < 0.001 and cluster-level p(FWE-corrected) < 0.05

Contrasting low versus high provocation did not yield any significant results.

##### Contrast aggression after high provocation versus aggression after low provocation

**Fig. 2:**
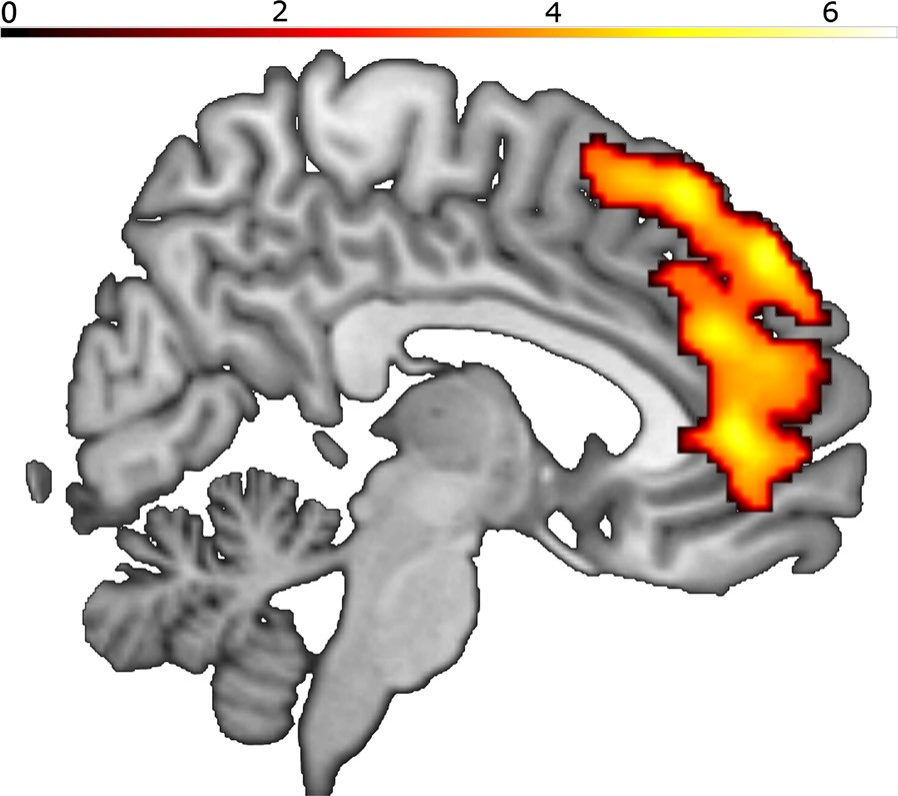
Aggression after high versus low provocation (all subjects): Sagittal view; x = 5; Activations for aggression contrast: rACC, mPFC, OFC. Color bar representing t-value

The contrast between aggression phases after high provocation versus aggression after low provocation revealed activation differences in the right mPFC, the left OFC, the right and left dlPFC, the right ventrolateral prefrontal cortex (vlPFC), the right rACC, and the left insula (Table 1; Fig. 2).

Contrasting aggression after low provocation versus aggression after high provocation did not yield any significant results.

For results for additional contrasts (win vs. lose trials) please see Additional file 1: Table S1 and Additional file 2: Figure S1.

#### Regression analysis with “average aggression” score

##### Contrast high provocation versus low provocation

**Fig. 3:**
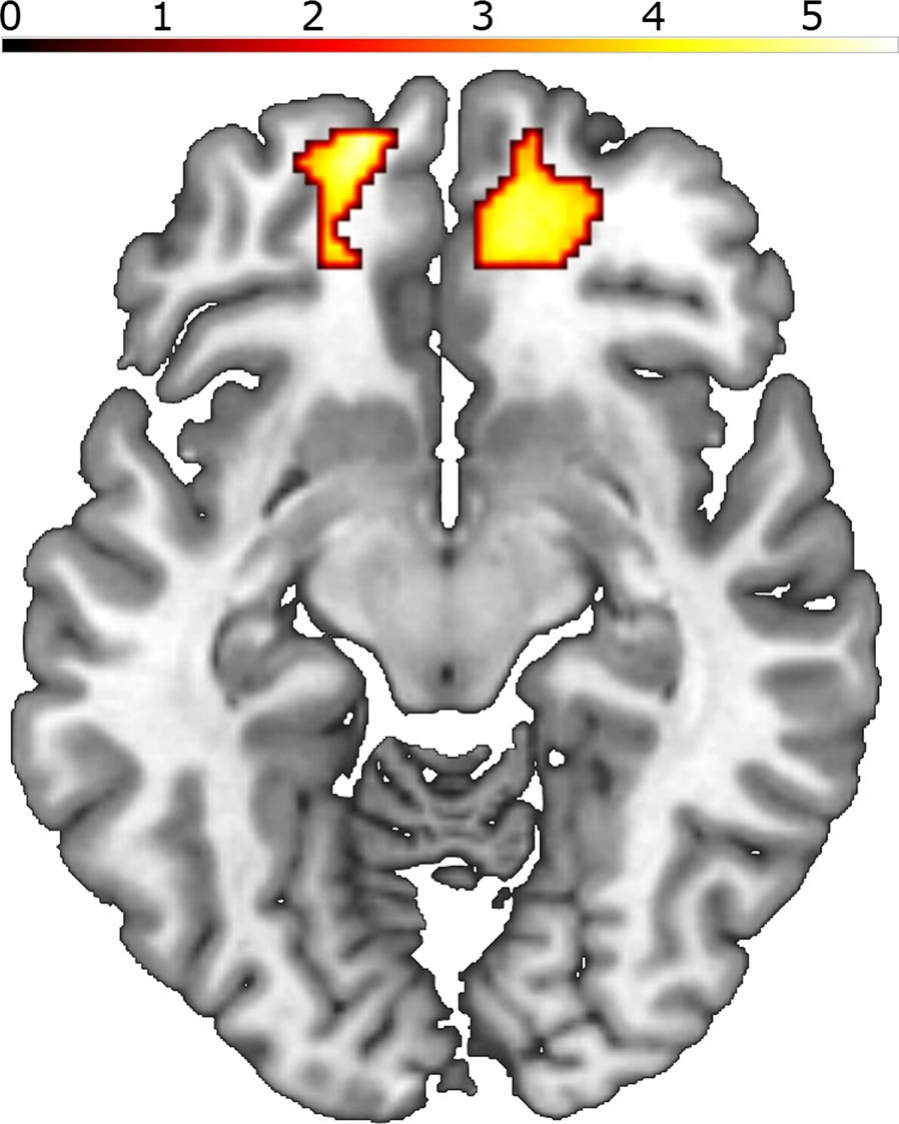
Association of “average aggression” score with the contrast high versus low provocation. Axial view; z = -10 bilateral association in the OFC, p < 0.001 and cluster-level p(FWE-corrected) < 0.05

The regression analysis between the contrast high versus low provocation and the “average aggression” (average punishment selection) score yielded a positive correlation with a cluster extending in the right superior and medial orbital gyrus and the right superior frontal gyrus and a cluster in the superior orbital gyrus (Table 2; Fig. 3).

**Table 2:** Association of “average aggression” score with the contrast high versus low provocation

Brain areas	L/R	X	Y	Z	T	Cluster size
		(MNI coordinates)				
Positive association with high versus low provocation						
OFC + mPFC	R	18	46	0	5.06	735
OFC	L	– 16	56	– 12	5.54	331
Negative association with high versus low provocation						
No suprathreshold activations						
Positive association with aggression after high versus low provocation						
No suprathreshold activations						
Negative association with aggression after high versus low provocation						
No suprathreshold activations						

Based on the SPM analysis with the contrast images “high versus low provocation” and the covariate “average aggression” score. Association of “Average aggression score” with contrast images high versus low provocation and aggression after high versus low provocation. p < 0.001 and cluster-level p(FWE-corrected) < 0.05

##### Contrast aggression after high provocation versus aggression after low provocation

This regression analysis did not yield any significant results.

Please see Additional file 3: Figure S2 for scatterplot illustration of association between activation difference and average aggression score.

### Correlational analyses

Correlation analyses between AQ scores, STAXI scores and extracted brain region activations from the provocation and aggression contrast did not reveal any significant correlations.

## Discussion

It is well-known that provocation can lead to aggressive responses. With a modified Taylor aggression paradigm we were able to show that this is reflected by activation of the brain’s aggression network already during the provocation phase. Hence, the direct influence of provocation on aggression is expressed by the preparation of the brain for an aggressive response. During the aggressive response itself the dorsal frontal control system comes into play. The extent of aggressive responses is further mediated by individuality.

### Provocation

Consistent with our hypotheses, behavioral data revealed significantly higher anger scores (STAXI, ESR) after the game than before. Taken together with higher punishment exerted after high than after low provocation, these data support the effectiveness of our aggression-provoking paradigm. By using a non-physical provocation technique that has proven to be effective in inducing aggressive behavior [8–10], the current experiment provides a good method for in vivo human aggression research under neuroimaging conditions.

The provocation phase stimulated the brain’s network implicated in aggression (Table 1). The rACC was active both in high (versus low) provocation as well as aggression after high (vs. low) provocation: ACC activity is thought to be attenuated in response to provocation in individuals prone to aggression [30], involved in negative emotion processing [31], volitional cognitive control, emotional salience and (together with the insula) exerts control over the autonomic nervous system [32, 33]. A further subdivision of the ACC has been established [34, 35]: Whereas the rostral part is involved in the regulation of acute affective arousal conveyed by the limbic system, angry rumination [36] and reward frustration [37], the dorsal part is active in appraisal and expression of negative emotion [5, 32, 38]. Therefore activation of rACC can be due to different processes. Most compelling in this context seems its role in the expression and regulation of affective arousal.

Similar to the ACC, the medial prefrontal cortex (mPFC) was active both in provocation and aggression. It has been linked to decision making [39] and the appraisal and expression of negative emotions [5]. Our data are consistent with the reactive aggression study by Lotze et al. [14], where the mPFC correlated with the intensity of the revenge stimulus the subjects administered. Specifically, this activation (especially in the aggression contrast) can be interpreted as the rising conflict between retaliation and non-aggressive responses and thus implicates the involvement of the mPFC in conflicting decision making.

### Aggression

In addition to mPFC and ACC activation, we found insula activation in the subsequent aggression contrast. The insula is described as part of the “negative brain”, relevant for the processing of negative stimuli [31], emotional pain, frustration [40], empathy [41] and social pain [42]. Most importantly, the insula (together with subcortical structures including hypothalamus and amygdala) is considered to be a part of a neural system that underlies the experience of aggressive impulses and emotion expression [2] and is involved both in reactive aggression and motor impulsivity [13, 17]. In contrast to these emotional “bottom-up drives” higher OFC activation (also in the aggression contrast) might be indicative of a stronger control attempt [2, 31] such that the OFC is involved in emotional decision making [43], frustration [44] and the suppression of negative emotions [31]. The OFC has been shown to suppress amygdala activity, which is increased in reactive aggressive populations (Intermittent Explosive Disorder; Spouse abusers) [7].

### The influence of individuality on aggression

Similarly, heightened activation in the OFC in the provocation contrast in more aggressively reacting individuals (regression analysis) might indicate the attempt to regulate an angry and aggressive response [2, 30]. This finding is in contrast to the simplistic model of top-down OFC control of aggressive impulses. This top-down model is supported by fMRI-studies where subjects imagined aggression [45, 46], and by lesion studies that show that patients with OFC lesions have higher aggression and violence scores compared to controls and patients with lesions in other brain regions [4]. Further support comes from a study demonstrating an inverse relationship between OFC reactivity to angry faces and aggressive responses in a TAP [47]. Another fMRI study pointed to attenuated OFC reactivity to provocation that correlated with increased aggressive responses [48].

In contrast to that, we demonstrated a positive correlation of aggressive responses and OFC activity during provocation and propose an intermediate role for the OFC in the aggression network: stronger OFC activity in this provocation contrast may reflect the stronger need for regulating aggressive impulses in participants exhibiting more aggressive responses [49]. Alternatively, the positive association between aggressive responses and OFC activity could be interpreted as the potential pleasure from punishing an unfair rival [50] in more aggressive subjects or increased compassion and empathy in those individuals with higher punishment selections [14].

These activation differences support the hypothesis that there is an association between aggressive retaliation and prefrontal response to provocation. Therefore, the increased aggressive behavior may be interpreted as being triggered by a higher susceptibility to provocation and not necessarily by a dysfunctional inhibition during the aggression phase, giving further support to the proposed connection between trait anger and reactive aggression [51, 52].

## Limitations

Disentangling provocation and aggression effects is challenging in such experimental settings, as they may be confounded. On average, high provocation leads to higher aggression and as these phases (feedback phase, punishment selection phase) are chronologically adjacent in our paradigm, it cannot be excluded that BOLD responses add up, exaggerating the observed provocation effects. Therefore differences between these two phases need to be interpreted with caution.

Other factors inherent to the design of this study could have contributed to a bias in the participants. Subjects filled in STAXI reports before the games, which for some could have led them towards the true purpose of the game instead of the alleged reaction time task.

## Conclusion

This study aimed to measure brain activity in an in vivo real-time provocation-aggression situation and showed multiple brain networks that are associated with those constructs. Future studies could apply this paradigm to investigate the neural mechanisms underlying aggressive behavior in psychiatric and neurological diseases. Our results contribute to (1) a more precise characterization of the involved brain circuits in *non*-*physical* provocation and aggression (compared to other aggression studies) and (2) highlight the crucial role of initial appraisal of provocation for subsequent regulatory and determinative function of emotional outcomes, aggressive behavior and the underlying aggression-related brain circuits.

## Additional files

**Additional file 1: Table S1.** Activations for whole sample of all subjects. Contrasts win vs. lose trials.

**Additional file 2: Figure S1.** Win vs. lose (all subjects): coronal view; y= 8; activations for contrast win > lose. Color bar representing t-value.

**Additional file 3: Figure S2.** Scatterplot showing strength of relationship between the average aggression score (average punishment selection) and the activity in the significant cluster in the OFC. (extracted from the contrast high vs. low provocation).

## Abbreviations

ACC: anterior cingulate cortex
AQ: Buss–Perry Aggression Questionnaire
dlPFC: dorsolateral prefrontal cortex
ESR: emotional self rating
FWE: family-wise error
GLM: general linear models
mPFC: medial prefrontal cortex
OFC: orbitofrontal cortex
PSAP: Point Aggression Subtraction Task
PANAS: Positive and Negative Affect Schedule
TAP: Taylor aggression paradigm
mTAP: modified version of the Taylor aggression paradigm
rACC: rostral anterior cingulate cortex
STAXI: State-Trait Anger Expression Inventory
SCID: Structural Clinical Interview for DSM Disorders
vlPFC: ventrolateral prefrontal cortex
