# Supplementary material for: From provocation to aggression: the neural network
Source: BMC Neurosci. 2017 Oct 17;18:73. doi: 10.1186/s12868-017-0390-z (PMC5646154; doi:10.1186/s12868-017-0390-z)
Supplement: Supplementary file 1 — Additional file 1: Table S1. Activations for whole sample of all subjects. Contrasts win vs. lose trials. [file 12868_2017_390_MOESM1_ESM.docx]

**Table S1. Activations for whole sample of all subjects. Contrasts win vs. lose trials**

| **Brain areas** | **L/R** | **X** | **Y** | **Z** | **T** | **p-value** | **Cluster size** |
| --- | --- | --- | --- | --- | --- | --- | --- |
|  |  | (MNI coordinates) | | |  |  |  |
| **win > lose** | | | | | | | |
| caudate nucleus/ mPFC/  olfactory cortex | R/L | 12 | 10 | -7 | 13.39 | <0.001 | 26753 |
| cuneus/ calcarine gyrus/ lingual gyrus | R | 40 | -64 | -33 | 6.99 | <0.001 | 2138 |
| lingual gyrus | L | -14 | -70 | -16 | 4.49 | <0.001 | 624 |
| superior temporal gyrus | R | 66 | -14 | 0 | 7.45 | <0.001 | 496 |
| cerebellum | L | -56 | -52 | -40 | 5.15 | <0.001 | 315 |
| supramarginal gyrus | R | 60 | -44 | 42 | 4.12 | <0.001 | 205 |
| **lose vs. win** | | | | | | | |
| superior medial gyrus | R | 6 | 48 | 36 | 5.76 | <0.001 | 295 |
| inferior frontal gyrus p. triangularis | R | 56 | 28 | 22 | 5.41 | <0.001 | 278 |
| temporal pole/entorhinal cortex | R | 36 | 20 | -28 | 4.88 | <0.001 | 224 |
| inferior frontal gyrus p. opercularis | R | 42 | 12 | 34 | 4.67 | <0.001 | 194 |

Activation cluster of all subjects, p<0.001 and cluster-level p(FWE-corrected)<0.05
