# Supplementary material for: From provocation to aggression: the neural network
Source: BMC Neurosci. 2017 Oct 17;18:73. doi: 10.1186/s12868-017-0390-z (PMC5646154; doi:10.1186/s12868-017-0390-z)
Supplement: Supplementary file 2 — Additional file 2: Figure S1. Win vs. lose (all subjects): coronal view; y= 8; activations for contrast win > lose. Color bar representing t-value. [file 12868_2017_390_MOESM2_ESM.docx]

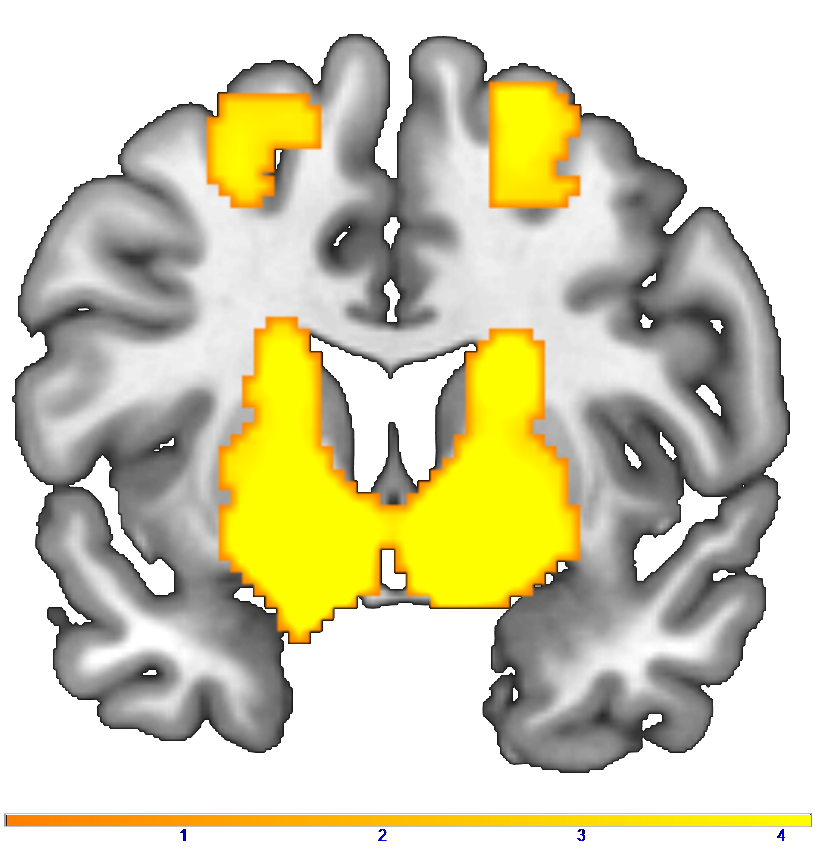


**Figure S1. Win vs. lose (all subjects):**

Coronal view; y= 8; Activations for contrast win > lose. Color bar representing t-value.
