# Supplementary material for: From provocation to aggression: the neural network
Source: BMC Neurosci. 2017 Oct 17;18:73. doi: 10.1186/s12868-017-0390-z (PMC5646154; doi:10.1186/s12868-017-0390-z)
Supplement: Supplementary file 3 — Additional file 3: Figure S2. Scatterplot showing strength of relationship between the average aggression score (average punishment selection) and the activity in the significant cluster in the OFC. (extracted from the contrast high vs. low provocation). [file 12868_2017_390_MOESM3_ESM.docx]

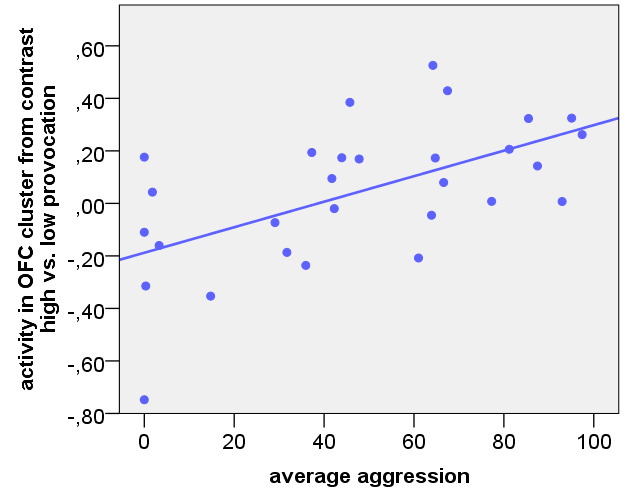


**Figure S2**. Scatterplot showing strength of relationship between the average aggression score (average punishment selection) and the activity in the significant cluster in the OFC. (extracted from the contrast high vs. low provocation).
